# Improving the Care of Children and Young People (CYP) Admitted to Adult Mental Health Psychiatric Beds in NHS Tayside Using Quality Improvement Methodology

**DOI:** 10.1192/bjo.2022.322

**Published:** 2022-06-20

**Authors:** Joy Olver

**Affiliations:** NHS Tayside, Dundee, United Kingdom

## Abstract

**Aims:**

To reduce monthly bed days for children and young people (CYP) aged under 18 years admitted to adult psychiatric beds by 50%

**Methods:**

QI tools used included driver diagram, stakeholder analyses, process mapping, ishikawa diagram, pareto chart and interviews with CYP and carers to gather qualitative data. Monthly data were collected on all admissions of CYP to adult mental health beds. Change ideas/ process changes included:

- Early senior psychiatric CAMHS review for all CYP admitted to adult psychiatric beds (same or next working day)
- Increased access to CAMHS medical records for out of hours staff
- Admission of all appropriate under 16's to paediatric beds instead of adult mental health beds
- Short test of change of staffing CAMHS specialist nurses over a weekend
- Develop alternative non-health crisis support/bed for CYP
- Develop Personality Disorder (PD) pathway

**Results:**

- Early senior CAMHS psychiatric review was associated with a reduction in CYP admitted to adult mental health beds from a median of 20 days a month to 2 days a month without an associated increase in CAMHS inpatient admissions
- Pareto chart showed that Personality Disorder (PD) was the commonest diagnosis
- Access to CAMHS medical records for all out of hours psychiatric medical staff was increased from 13% to 100%
- Routine admission to paediatrics for all under 16's was agreed with paediatric medical and nursing managers but not sustainably implemented
- There were no acute referrals to the CAMHS specialist nurses over the single weekend short test of change
- Development of an alternative non-health crisis support/bed and development of a Personality Disorder (PD) pathway is still in process

**Conclusion:**

The primary outcome measure was successfully met with the median bed days of CYP admitted to adult mental health beds sustainably reduced from a median of 20 days to 2 days. This was associated with the implementation of routine early senior psychiatric CAMHS review and increased access to CAMHS health records for all medical staff providing psychiatric out of hours assessments. The change ideas including development of different admission pathways (paediatrics and non-health crisis bed), weekend CAMHS specialist nurses service and development of a personality disorder pathway were not implemented sustainably. The pathways of care around CYP presenting in crisis are complex. Making sustainable improvements in complex adaptive systems is complex and challenging but not impossible.

